# Crystal Structure and Inhibitor Identifications Reveal Targeting Opportunity for the Atypical MAPK Kinase ERK3

**DOI:** 10.3390/ijms21217953

**Published:** 2020-10-26

**Authors:** Martin Schröder, Panagis Filippakopoulos, Martin P. Schwalm, Carla A. Ferrer, David H. Drewry, Stefan Knapp, Apirat Chaikuad

**Affiliations:** 1 Structural Genomics Consortium, Goethe University Frankfurt, Buchmann Institute for Molecular Life Sciences, Max-von-Laue-Straße 15, 60438 Frankfurt am Main, Germany; m.schroeder@pharmchem.uni-frankfurt.de; 2 Institute of Pharmaceutical Chemistry, Goethe University Frankfurt, Max-von-Laue-Straße 9, 60438 Frankfurt am Main, Germany; schwalm@pharmchem.uni-frankfurt.de; 3 Structural Genomics Consortium, Nuffield Department of Medicine, University of Oxford, Old Road Campus Research Building, Roosevelt Drive, Oxford OX3 7DQ, UK; panagis.filippakopoulos@cmd.ox.ac.uk; 4 Structural Genomics Consortium, UNC Eshelman School of Pharmacy, University of North Carolina, Chapel Hill, NC 27599, USA; carla.alamilloferrer@manchester.ac.uk (C.A.F.); david.drewry@unc.edu (D.H.D.); 5 German Cancer network DKTK and Frankfurt Cancer Institute (FCI), Goethe University Frankfurt, 60438 Frankfurt am Main, Germany

**Keywords:** atypical MAPK kinase, ERK3, MAPK6, kinase inhibitors, covalent inhibitors

## Abstract

Extracellular signal-regulated kinase 3 (ERK3), known also as mitogen-activated protein kinase 6 (MAPK6), is an atypical member of MAPK kinase family, which has been poorly studied. Little is known regarding its function in biological processes, yet this atypical kinase has been suggested to play important roles in the migration and invasiveness of certain cancers. The lack of tools, such as a selective inhibitor, hampers the study of ERK3 biology. Here, we report the crystal structure of the kinase domain of this atypical MAPK kinase, providing molecular insights into its distinct ATP binding pocket compared to the classical MAPK ERK2, explaining differences in their inhibitor binding properties. Medium-scale small molecule screening identified a number of inhibitors, several of which unexpectedly exhibited remarkably high inhibitory potencies. The crystal structure of CLK1 in complex with CAF052, one of the most potent inhibitors identified for ERK3, revealed typical type-I binding mode of the inhibitor, which by structural comparison could likely be maintained in ERK3. Together with the presented structural insights, these diverse chemical scaffolds displaying both reversible and irreversible modes of action, will serve as a starting point for the development of selective inhibitors for ERK3, which will be beneficial for elucidating the important functions of this understudied kinase.

## 1. Introduction

Protein kinases comprise one of the largest protein families, with more than 500 members [1]. Even though dysregulation of many protein kinases has been linked to the development of disease, only a small subset of kinases has been a focus of inhibitor and drug development, while a large proportion of this protein family remain understudied [2]. In order to explore the biology of understudied kinases, a number of research programs have been established aimed at elucidating the biology of the “dark” kinome, as well as other neglected human proteins [3,4]. Also, in recent years there has been a significant effort to elucidate the molecular functions of kinases and their roles in diseases, using chemical tools targeting the kinase family, such as the kinase inhibitor set PKIS (published kinase inhibitor) [5,6,7] and more recently the kinase chemogenomics set (KCGS) [8], as well as developed chemical probes [5,6,9].

Extracellular signal-regulated kinase 3 (ERK3) is a poorly studied member of the mitogen-activated protein kinase (MAPK) family [10]. This family contains 14 members, sharing structural features like the MAPK insertion signature located in the *C*-terminal lobe of the kinase domain [11,12]. Similar to ERK4, ERK7, and NLK, ERK3 is classified as an atypical MAPK [10]. Major differences when compared to the classical MAPKs include, for example, the substitution of the conserved threonine–x–tyrosine (TxY) motif within the activation segment by the ERK3 serine–glutamate–glycine (SEG) sequence, the alteration of the conserved alanine–proline–glutamate (APE) motif at the end of the activation segment to the unique serine–arginine–proline (SRP) motif, and the extended *C*-terminal tail harbouring the protein–protein interaction domain, called C34, that is present also in ERK4 [10,13]. Unlike other members, such as p38, ERK1/2 and c-Jun *N*-terminal kinases (JNKs), several lines of evidence have demonstrated that ERK3 does not follow the canonical MAPK activation mechanism, which comprises phosphorylation of the TxY motif by mitogen-activated protein kinase kinase (MAP2K) [14]. In contrast, phosphorylation of the serine in the ERK3 SEG motif is carried out by a non-MAPK; for instance, the p21-activated kinase 1 (PAK1) [15,16]. In addition, previous studies have suggested that unlike other classical MAPKs, phosphorylation and activation of this atypical MAPK are not influenced or triggered by mitogenic stimuli like sorbitol or hydrogen peroxide [17].

MAP kinases are key signalling molecules that are important in transducing extracellular signals, and thus regulate various cellular processes, including proliferation, differentiation, and cell survival [18]. However, little is known regarding the involvement of ERK3 in specific biological processes. Nonetheless, a few substrates of this atypical MAPK have been identified. This includes the well-validated MAPK-activated protein kinase 5 (MAPKAPK5, also called MK5) [19,20,21], as well as other potential downstream targets, such as steroid receptor coactivator 3 (SRC-3) [22], tyrosyl DNA phosphodiesterase 2 (TDP2) [23], and the septin-interacting protein binders of Rho GTPases 1–3 (BORGs) [24]. Thus, the potential functions and physiological importance of ERK3 stem from these activities. Genetic knock-out experiments of the MAPK6 gene encoding ERK3 lead to pulmonary immaturity and neonatal lethality in mice, highlighting its importance for pulmonary differentiation [25]. In addition, ERK3-mediated SRC-3 phosphorylation, leading to an increase of VEGFR2 expression levels, is important for controlling epithelial cell migration [26]. High levels of SRC-3 phosphorylation due to the upregulation of ERK3 is linked to an elevated level of MMP (matrix metalloprotease) gene expression, which in turn enhances invasiveness of lung cancer cells [22]. Furthermore, ERK3 phosphorylates and regulates the phosphodiesterase activity of TDP2, which has been shown to modulate DNA damage response in lung cancer cells, resulting in chemoresistance to topoisomerase2 inhibitors [23]. Moreover, ERK3 is also highly expressed in head and neck cancer [27] and breast cancer [28], suggesting that this kinase might play a role in the development of diseases.

Despite potentially important functions in cellular processes and tumorigenesis, no ERK3-selective inhibitors have been published that could be used as tools to elucidate the roles of this kinase in normal and pathological signalling processes. A few potential inhibitors of ERK3 have been detected in kinome-wide small molecule screening, such as PKIS studies [6,7] or large selectivity screens. For instance, the JNK inhibitor JNK-IN-7 has been reported to have ERK3 off-target activity [29].

To assist selective inhibitor development effort, we describe in this study the crystal structure of ERK3. Structural comparison suggested that in addition to the diverse activation segment, the ERK3 ATP binding site also harbours unique sequence variations, distinguishing it from other ERK family members. Screening of over 1400 inhibitors by temperature shift assays revealed a number of hits from diverse chemical scaffolds. We identified ERK3 inhibitors with both reversible and irreversible modes of action and confirmed their target engagement in cells. These structural insights, together with the identified inhibitors, provide molecular information and a valuable set of chemical starting points for further ERK3 inhibitor development efforts.

## 2. Results and Discussion

### 2.1. Crystal Structure of ERK3 Revealed Differences to the Classical MAPK ERK2

**Figure 1:**
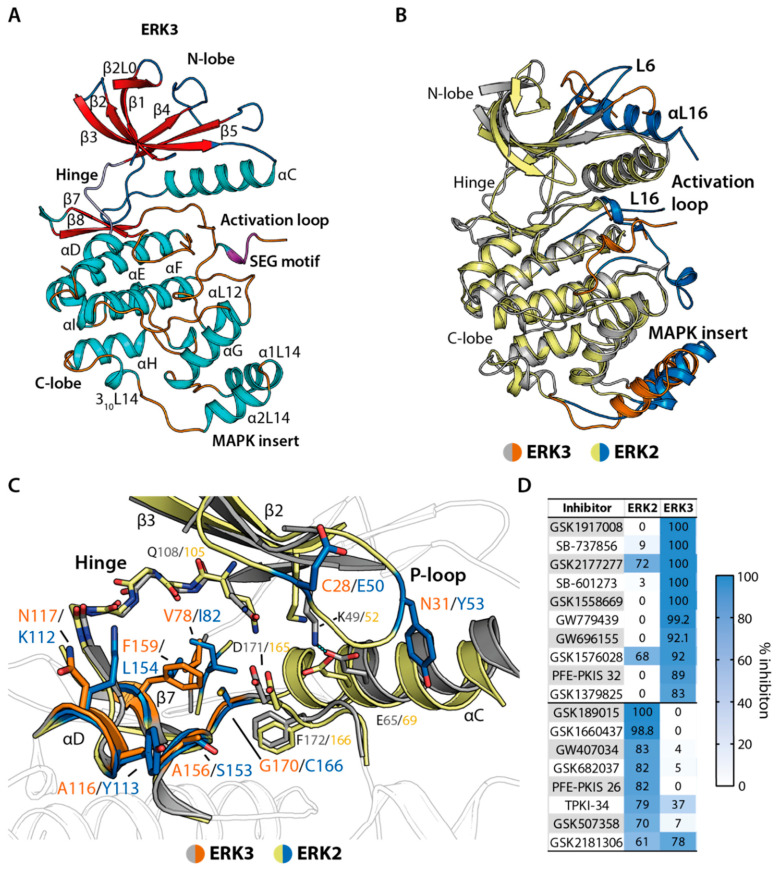
Crystal structure of extracellular signal-regulated kinase (ERK) 3 kinase domain and structural comparison with ERK2. (**A**) Crystal structure of the kinase domain of human ERK3 (PDB ID: 7aqb). Secondary structure elements are colour-coded, with α-helices shown in cyan and β-strands in red. (**B**) Superimposition of the kinase domains of ERK3 (grey) and ERK2 (yellow) (PDB ID: 5ngu [35]). Highlighted in orange (ERK3) and blue (ERK2) are the main differences between both structures. (**C**) Comparison of the ATP binding pockets of ERK3 (grey) and ERK2 (yellow). Amino acid differences between ERK3 (orange) and ERK2 (blue) are highlighted. (**D**) Examples of identified inhibitors for ERK2 and ERK3 from the published kinase inhibitor (PKIS) studies [6,7] reveal different inhibitor preferences between these two kinases.

The lack of structural information hinders our understanding of the characteristics of ERK3, and hence rational inhibitor development. Thus, we determined the crystal structure of the kinase domain of this atypical MAPK (Figure 1A). Overall, the kinase domain of ERK3 adopted the typical kinase topology, comprising the *N*-terminal lobe containing β-stands and the *C*-terminal lobe constructed mainly from helices. The two monomers in the asymmetric unit were observed to form a dimer in a head-to-tail, face-to-face fashion (Figure S1). However, we expected that this dimer was likely induced only in the crystals, as the kinase was observed to behave as a monomer in solution as determined by size exclusion chromatography (Figure S1). Detailed structural analyses revealed that, as expected for an unphosphorylated and ligand-free state, most parts of the activation loop and the glycine-rich loop (P-loop) of both ERK3 molecules in the asymmetric unit were disordered. Nevertheless, there were several features that displayed an active kinase conformation, including an “in” conformation of the tripeptide motif DFG (aspartate, phenylanaline, glycine), as well as the canonical salt bridge contact between the β3 K49 and E65 located in the αC helix.

In comparison, the overall structure of the kinase domain of ERK3 resembled that of the classical MAPK ERK2. Nonetheless, some small differences were observed for the loop connecting β 4 and β 5, the unphosphorylated activation segment, and the α1L14 and α2L14 located within the MAPK insert (Figure 1B). In addition, partly due to the shorter protein used in crystallization, our ERK3 structure lacked the *C*-terminal L16 region typically present in the classical MAPKs. Nevertheless, the *C*-terminal region of ERK3 is expected to be highly different, due to the existence of the C34 domain in this atypical MAPK.

Detailed structural analyses further demonstrated high diversity of the residues of ERK3 and ERK2 within the ATP binding pockets that typically engage in inhibitor binding (Figure 1C). Key differences include (i) the substitutions of the bulky Y113 and the basic K114, located in the αD helix of ERK2 to the smaller A116 and neutral N117 in ERK3, respectively; (ii) the change of ERK2 E50 to ERK3 C28 in the P-loop; and (iii) the alteration of the residue preceding the DFG motif (DFG-1 residue) from C166 in ERK2 to Gly170 in ERK3. The KLIFS database (https://klifs.net/index.php), as well as previous studies, has shown that the residues at these positions could control kinase sensitivity to inhibitors [30,31]. Indeed, analysis of the published PKIS2 dataset [6,7] revealed almost no inhibitors targeting both ERK2 and ERK3 (Figure 1D). This suggests, therefore, that different chemical scaffolds and potential targeting strategies are required for ERK3, of which the unique pocket would provide a basis for selectivity over ERK2.

### 2.2. Screening and Characterization of Inhibitors for ERK3

**Figure 2:**
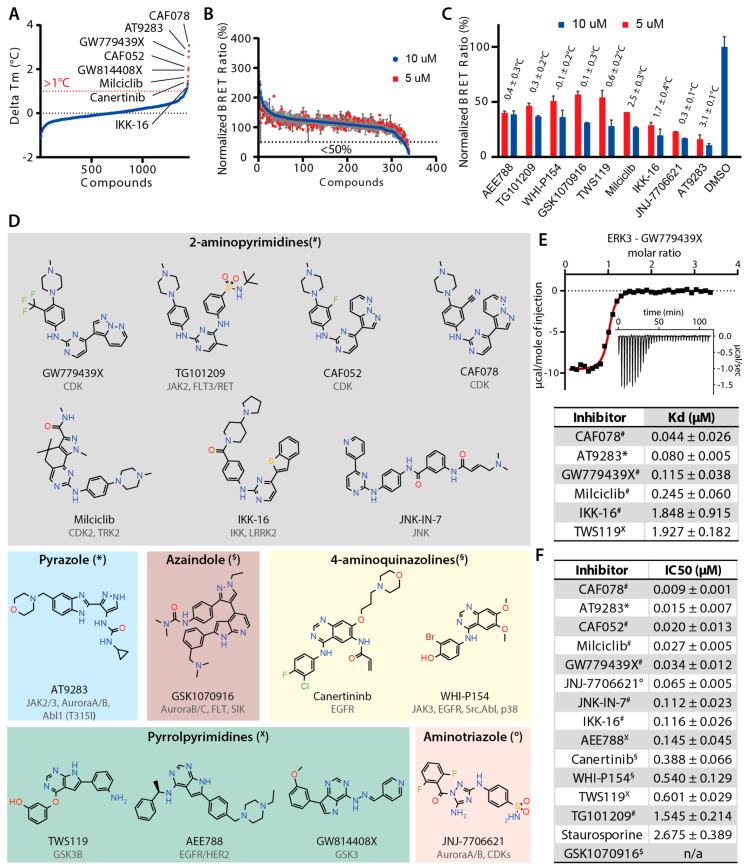
Identification of potential inhibitor ERK3. (**A**) Temperature shift results from inhibitor screening for ERK3. (**B**) The screening results from the NanoBRET assay. The normalized BRET ratio is calculated from the ratio between the BRET signal of the tested inhibitors and that of the DMSO control. The black dashed line indicates the normalized ratio at 50%. (**C**) Plots of the normalized BRET ratios for top hits (red and blue bars for 5 and 10 µM concentrations, respectively), with their measured temperature shift results displayed. (**D**) Chemical structures of the selected 13 inhibitors identified from both screenings, which are classified into six groups based on their hinge binding motifs. (**E**) Binding affinities of the inhibitors in ERK3 measured by isothermal calorimetry (ITC). Top: the binding isotherm of the ERK3-GW779439X titration (inset) and the integrated heat of binding, with the red line showing the fitting of single-site binding. Bottom: summary of ITC dissociation constant (*K_d_*) and thermodynamic parameters averaged over two replicates. (**F**) The NanoBRET IC_50_ values for the inhibitors averaged over two replicates.

To search for potential inhibitors for ERK3, we performed inhibitor screening using two orthogonal assays. First, we screened an in-house library of 1454 kinase inhibitors, which included the PKIS set [6,7], using a differential scanning flourimetry (DSF) assay [32]. However, most inhibitors disappointedly showed only low melting temperature shifts (ΔTm), with the best three hits exhibiting ΔTm values of only 3.1–3.8 °C, and only 32 compounds with a ΔTm of >1 °C (Figure 2A and Table S2). Such low ΔTm values generally indicates weak or no binding of inhibitors in protein kinases [32]. Nevertheless, we speculated that a high melting temperature of 58 °C of ERK3 might limit the sensitivity of this assay to distinguish potential inhibitors that interacted with this kinase. Thus, we performed an orthogonal screening, using a cell-based NanoBRET assay [33,34] with a smaller library of 339 published kinase inhibitors, of which 337 were tested in the DSF assay. The results revealed that 14 compounds were capable of reducing the bioluminescence resonance energy transfer (BRET) signal by >50% at 10 µM, indicating their abilities to displace more than half fractions of the bound assay tracer from the kinase (Figure 2B and Table S3). We observed that among the hits, six were likely more potent and exerted similar activities at 5 µM. Interestingly, some of the hits from the NanoBRET screening exhibited almost no stabilisation effect in the DSF assay (Figure 2C).

Considering the results from both screening efforts, we selected the top nine hits from the NanoBRET screening, three hits from the DSF assay that were not included in the NanoBRET assay, and JNK-IN-7, previously showed to interact with ERK3 [29], for further characterisation. These 13 inhibitors could be classified into six groups based on their hinge binding moieties (Figure 2D): (i) aminopyrimidine (STK1 inhibitor GW779439X [36], CAF052 and CAF078 [37], IKK/LRRK2 inhibitor IKK-16, JAK inhibitors TG101209, Milciclib, and JNK-IN-7 [29]); (ii) pyrazole–bezimidazole (AT9283 that target JAK2/3, Aurora A/B, and ABL1 T315I); (iii) azaindole (Aurora B/C inhibitor GSK1070916); (iv) 4-aminoquinazoline (Canertinib and WHI-P154); (v) pyrrolopyrimidine (TWS119, AEE788, and GW814408X [7,38]); and (vi) aminotriazole (JNJ-7706621). To assess their binding affinities, we first used isothermal calorimetry (ITC) for a subset of the identified inhibitors, from which the results confirmed their interactions with the kinase, albeit by different degrees (Figure 2E and Figure S2B). By comparison, the ITC dissociation constant (*K_d_*) correlated with the ΔTm values; low nanomolar affinities were evident for CAF078 and AT9283, which exhibited the highest ΔTm of ~3.1–3.8 °C, whereas the higher *K_d_* values were measured for the inhibitors that showed lower ΔTm values of less than 2 °C. Next, in order to assess inhibitor binding more broadly, we determined the IC_50_ of all 13 inhibitors using the cell-based NanoBRET assay (Figure 2F and Figure S2C). Nearly all inhibitors, except for TG101209 and GSK1070916, the latter of which did not produce a BRET signal, exhibited nanomolar affinities. Remarkably strong inhibitory potencies (IC_50_ values of 9–65 nM) were detected for several aminopyrimidine inhibitors, including CAF078, AT9283, CAF052, Milciclib, and GW779439, as well as aminotriazole JNJ-770662. Taken together, both characterizations consistently confirmed the interactions of these inhibitors in ERK3, and the trend of the measured affinities correlated well with CAF078, AT9283, GW779439X, CAF052, and Milciclib, identified as the most potent inhibitors for the kinase.

The results from inhibitor screening provide evidence that this atypical MAPK kinase ERK3 can be targeted by diverse kinase inhibitors that target the ATP binding site. Interestingly, our screen revealed that most inhibitors binding to ERK3 were designed for kinases outside the MAPK family, coinciding with the selectivity pattern observed in the PKIS screening [6,7]. High diversity of the identified hits suggests that the ATP binding pocket of ERK3 can accommodate various hinge binding motifs with different decorations.

### 2.3. Irreversible Inhibitors Interacted with ERK3 via Covalent Targeting of the β1 Cysteine

**Figure 3:**
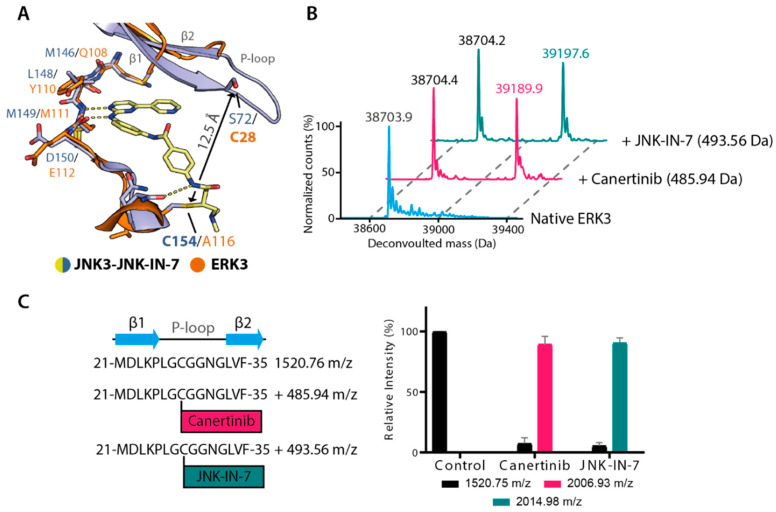
Irreversible binding of canertinib and JNK-IN-7 in ERK3. (**A**) Superimposition of the JNK3-JNK-IN-7 complex and ERK3 structure. (**B**) Intact mass analyses indicate covalent adducts between ERK3 and canertinib or JNK-IN-7. The counts of the highest peaks in each spectrum were normalized to 100%. (**C**) Mass spectrometry analyses of the peptides after chymotrypsin digestion of ERK3, which was pre-incubated with canertinib or JNK-IN-7 for 3 h. Left: the sequence of the digested peptide that covers the glycine rich loop region harbouring C28. The *m*/*z* shifts of +485.94 and +493.56 suggest C28 residue in this peptide is the target for covalent bonding with canertinib and JNK-IN-7, respectively. Right: relative intensity of the peptides from mass-spectrometry analysis.

Among the identified inhibitors, canertinib and JNK-IN-7 were particularly interesting hits. These inhibitors exert an irreversible mode of binding to their main targets, EGFR [39] and JNK3 [29], respectively, via a covalent bond with a cysteine residue located within the αD (Figure 3A). Although ERK3 does not possess an amino acid at this positions, previous analyses have predicted that the β1 C28 of this kinase might be accessible for covalent interaction with irreversible inhibitors [40]. Unfortunately, due to the disordered P-loop, our crystal structure of the kinase could not provide the exact position and conformation of this cysteine. To predict potential covalent binding of, for example, JNK-IN-7 in ERK3, we thus performed structural comparison against JNK3. With ~12.5 Å distance between the nucleophlilic warhead of the inhibitor and the side chain of JNK3 S72, the residue corresponding to ERK3 C28, we hypothesized that a rotation of the inhibitor around its amide group linking two phenyl moieties might possibly position the warhead for covalent contact with ERK3 β1 C28 (Figure 3A).

To test potentially irreversible binding of canertinib and JNK-IN-7 in ERK3, we incubated the kinase with the inhibitors at a 1:1 ratio for 30 min, and analysed the covalent adducts using mass spectrometry. Indeed, we observed the mass shifts indicating covalent formation, albeit only partially under this reaction condition (Figure 3B). To further confirm whether both inhibitors targeted C28, we generated the complete adducts and performed chymotrypsin digestion, followed by mass spectrometry analyses, from which the results confirmed C28 as the targeted residue (Figure 3C and Figure S3). Our observation opened an alternative opportunity for targeting ERK3 with irreversible inhibitors, as well provided evidence for the potential targeting β1 cysteine residues in other kinases that are scarce across the kinome [40]. Nevertheless, challenges remain due to flexibility within the linker region between its hinge binding moiety and its nucleophilic warhead, observed in both canertinib and JNK-IN-7. In order to selectively target the glycine-rich loop cysteine of ERK3, a more rigid scaffold would be required to prevent alternative orientations leading to unwanted covalent bond formation, as exemplified here by the JNK inhibitor JNK-IN-7 and the EGFR inhibitor canertinib.

### 2.4. Structural Comparison Provided Insights into Potential CAF052 Binding Mode in ERK3

**Figure 4:**
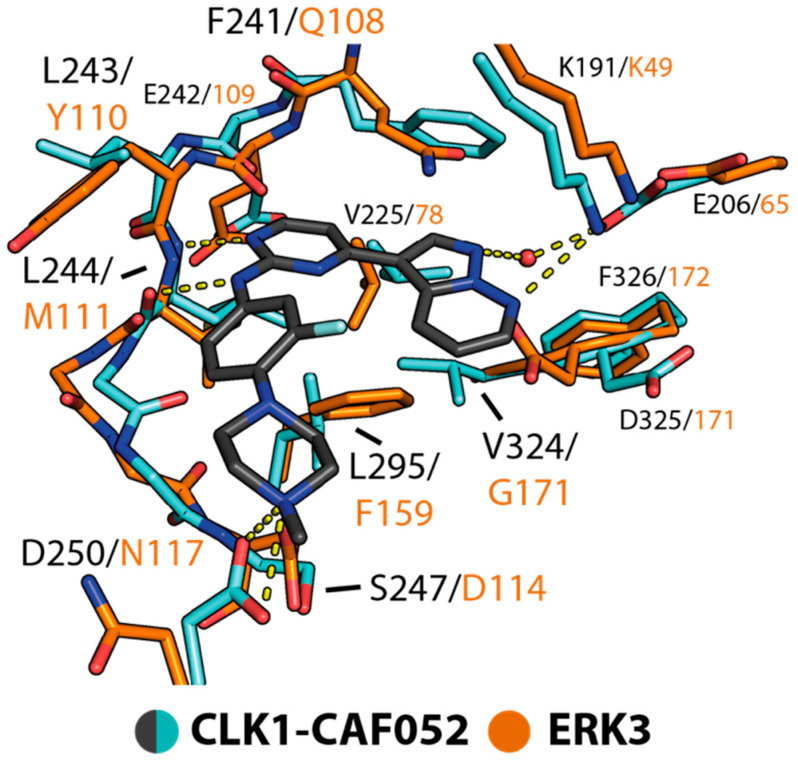
Structural comparison of binding of CAF052 in CDC-like kinase 1 (CLK1) and ERK3. Superimposition of the CLK1–CAF052 complex (PDB 7ak3) and ERK3 suggests potentially similar binding modes of the inhibitor in both kinases. Contacts observed between the inhibitor and CLK1 are shown with yellow dashed lines.

In order to provide structural insights into inhibitor binding, we attempted to determine the complex structure of ERK3 and inhibitors. Unfortunately, we did not succeed in obtaining crystals of any complexes, either by co-crystallization or soaking. Therefore, we determined the structure of CAF052 in complex with CDC-like kinase 1 (CLK1), which belongs to the same kinase group (CMGC) as ERK3, and performed structural comparison (Figure 4). In CLK1, CAF052 adopted a typical type-I binding mode, utilizing its 2-aminopyrimidine core for the interactions with the backbone of the hinge residue L244. The observed binding mode of the inhibitor was highly similar to that of the chemically closely-related compound GW807982X in CLK1 (PDB ID: 6zln) [31] (Figure S4), and to the predicted binding modes of the related pyrazolo[1,5-b]pyridazines in GSK3B and CDK2 [41]. This suggested therefore that this binding orientation of CAF052 in CLK1 might be shared across diverse kinases. Structural superimposition demonstrated that although sharing a low-sequent identity (Figure S5) the binding mode of the inhibitor, as well as the contacts with the hinge region observed in CLK1, could most likely be maintained in ERK3 (Figure 4). Nevertheless, other interactions observed in CLK1 might differ in ERK3 due to amino acid variations. This included the contact between the inhibitor piperazine and the αD D250 in CLK1, which might likely be absent in ERK3 harbouring N117 at this position. In this binding mode, the pyrazolopyridazine group was expected to still bind within the back pocket of ERK3 and form a contact with the β3 catalytic lysine. Nonetheless, other interactions engaged by this moiety were presumably different, due to the substitutions of a F241 gatekeeper and V324 in CLK1 for Q108 and G171, respectively, in ERK3. This structural comparison therefore suggests that ERK3 likely shares similar mechanisms for accommodating inhibitors to that of other kinases, enabling binding of various inhibitors that are developed for protein kinases from diverse families. Nonetheless, detailed interactions would be highly expected, due to differences within the ATP binding pocket.

## 3. Discussion

“Untargeted” kinases have recently received more attention, as understanding their roles in biological processes and their involvement in the development of diseases would open opportunities for novel therapies. However, studying the functions of these dark kinases remains challenging, due to a lack of tools, such as selective inhibitors and so-called chemical probes [42,43]. The development of such tool compounds often requires detailed structural knowledge of the binding pocket, as well diverse chemical starting points to facilitate achieving the required potency and selectivity for their targeted kinase, which is usually challenging considering the large size of the family [42].

To contribute to recent global research efforts on untargeted human proteins [44], we present the crystal structure as well as the discovery of inhibitors of ERK3, providing a foundation for the development of selective inhibitors for this poorly-studied, atypical MAPK kinase. Our screening identified several diverse inhibitors that can interact with ERK3, which is rather unexpected, providing previous little reports on inhibitors for this kinase. Interestingly, most hits were inhibitors that were developed for kinases outside the MAPK family, yet many, such as Aurora inhibitor AT9283 and STK1 inhibitor CAF078, surprisingly exhibited high inhibitory potencies in a low nanomolar range for ERK3. In addition, our observation on the irreversible binding of canertinib and JNK-IN-7 is intriguing, and coincides with a previous prediction on the accessibility of the β1 C28 for covalent inhibitors [40].

ERK3 is classified as an atypical MAPK, and harbours several distinct features compared to the classical MAPKs [10,13]. Consistently, the presented crystal structure revealed several differences between the ATP binding pocket of this atypical kinase and that of the classical MAPK ERK2, which is in agreement with our analyses of the PKIS dataset [6,7] revealing almost no common inhibitors between these two kinases. However, the crystal structures of the inhibitor-bound complexes are still required to delineate the contribution of amino acid variations in ERK3 towards its different sensitivity toward inhibitors, which could potentially provide a basis for isoform selectivity.

In summary, the identified inhibitors, with both reversible and irreversible modes of action, open an opportunity for targeting this atypical ERK3. Together with the presented structural insights, these chemical starting points will be beneficial for the development of potent and selective inhibitors, which will serve as a powerful tool for elucidating the biological roles of this understudied MAPK.

## 4. Materials and Methods

### 4.1. Protein Expression and Purification

The kinase domain of ERK3 (M9-I327) was subcloned into pNIC28–Bsa4, and the recombinant protein containing an *N*-terminal His_6_ tag was co-expressed with lambda phosphatase in *E. coli*. Cells were harvested, resuspended in 50 mM 4-(2-hydroxyethyl)-1-piperazineethanesulfonic acid (HEPES) pH 7.5, 500 mM NaCl, 30 mM imidazole, 1 mM tris(2-carboxyethyl)phosphine (TCEP), and 5% glycerol supplemented with a protease inhibitor cocktail (Merck, Darmstadt, Germany), and subsequently lysed by sonication. After centrifugation at 60,000× *g* for 30 min at 4 °C, the recombinant protein in the supernatant was purified by Co^2+^-affinity chromatography. Tobacco etch virus (TEV) protease was added to remove the *N*-terminal affinity tag, and the cleaved ERK3 protein was further purified by reverse Ni^2+^-affinity chromatography. The recombinant protein was further purified using Superdex S75 column (GE healthcare, Chicago, IL, USA) in 10 mM HEPES pH 7.5, 100 mM NaCl, and 10 mM dithiothreitol (DTT). For CLK1, the recombinant kinase domain (H148-I484) was co-expressed with lambda phosphatase in *E. coli*. The protein was purified according to protocol published previously [31,45].

### 4.2. Structure Determination

Crystallization were performed at 4 °C, using the sitting drop vapour diffusion method with ERK3 at 20 mg/mL and the reservoir containing 1.8 M MgSO_4_ and 0.1 M 2-(*N*-morpholino)ethanesulfonic acid (MES) pH 6.7. Crystals were cryoprotected with mother liquor supplemented with 20% ethylene glycol. Diffraction data were collected at SLS beamline X10SA. For the CAF052–CLK1 structure, the complex was prepared by mixing the protein at 8 mg/mL with the inhibitor at 1 mM, and crystallization of the complex was performed using the sitting drop vapour diffusion method at 4 °C. The crystals were obtained using the reservoir solution containing 23% polyethylene glycol (PEG) 6000 and 0.1 M bicine pH 9.3. The crystals were cryoprotected with mother liquor supplemented with 25% ethylene glycol, and diffraction data were collected at Diamond Light Source, beamline I04.

Diffraction data were processed with XDS [46], and scaled using aimless [47]. Molecular replacement was performed with Phaser [48] and the coordinates of ERK1 (PDB code: 1erk [49]) for ERK3 or CLK1 (PDB code: 1z57 [50]). Rounds of model building alternated with structure refinement were performed in COOT [51] and REFMAC [52], respectively. Model geometry was validated by MolProbity [53]. Data collection and refinement statistics are summarized in Table S1. The coordinates and structure factors of the crystal structures are available from the PDB under accession codes 7aqb and 7ak3.

### 4.3. Melting Temperature Assay (Differential Scanning Flourimetry (DSF))

The ERK3 kinase domain at 2 µM in 10 mM HEPES pH 7.5 and 500 mM NaCl was mixed with 10 µM inhibitors and SyPRO Orange at 1:1000 dilution. After 10 min incubation at room temperature, the fluorescence signal, which increases upon the temperature-dependent unfolding of ERK3 protein, was measured using Mx3005p real-time PCR machine (Stratagene, San Diego, CA, USA). The ∆Tm shifts were calculated using protocol described previously [32].

### 4.4. NanoBRET

Full length ERK3 was cloned into pFC32K (Promega, Madison, WI, USA) for the expression of an *N*-terminal NanoLuc fusion, and the plasmid was transfected into HEK293T cells cultured in DMEM (Gibco) supplemented with 10% fetal bovine serum (Gibco) and penicillin/streptamycin (Gibco, Thermo Fisher Scientific, Waltham, MA, USA). NanoBRET assays were performed using protocol published previously [54]. Briefly, after transfection and 20 h incubation, cells were harvested and subsequently resuspended in OptiMEM (Gibco). Cells were aliquoted onto 1534-well plates (Greiner Bio-One, Kremsmünster, Austria), and inhibitors and 0.25 µM Tracer K5 (Promega) were added using an ECHO acoustic dispenser (Labcyte, San Jose, CA, USA). The plates were incubated at 37°C with 5% CO_2_ for 2 h prior to the additions of both NanoBRET Nano-Glo substrate (Promega) and extracellular NanoLuc inhibitor. BRET luminescence (450 nm for donor emission and 610 nm for BRET signal) was measured using PHERAstar FSX plate reader (BMG Labtech, Ortenberg, Germany). Milli-BRET units (mBU) were calculated as a ratio between the BRET signal and the overall measured luminescence. A dose–response fitting was applied, and the corresponding IC_50_ values were calculated in Prism software. For the single concentration screening, the normalized BRET values were calculated from the ratio between the BRET signals of the tested inhibitor and that of the DMSO control.

### 4.5. Isothermal Titration Calorimetry

All ITC measurements were performed using NanoITC (TA Instruments, New Castle, DE, USA) at 15 °C. The ERK3 kinase domain in 20 mM HEPES, pH 7.5, 200 mM NaCl, 0.5 mM TCEP, and 5% glycerol at 180 µM was titrated into the reaction cell containing the inhibitors at 0.05—20.00 µM. The integrated heat of titration was calculated and fitted into an independent binding model using NanoAnalyzer software. Thermodynamic parameters, the dissociation constant (*Kd*), and stoichiometry (*n*) were an average over duplicates.

### 4.6. Mass Spectrometry Analyses

The recombinant kinase domain of ERK3 at 100 µM was incubated with equimolar canertinib, JNK-IN-7, or DMSO at room temperature for 30 min. The protein was purified using C8 stage tips [55], and the intact mass was analysed using LC-ESI TOF (Agilient, Santa Clara, CA, USA) and Bioconfirm software (Agilient). Chymotrypsin digestion followed by mass spectrometry analysis was performed to identify the residue that forms a covalent bond with the inhibitors. In brief, the recombinant ERK3 at 50 µM was incubated with 100 µM canertinib or JNK-IN-7 for 4 h at room temperature. The protein was subsequently precipitated by 50% trifluoroacetic acid, and the resulting pellet was washed twice with cold acetone. Chymotrypsin (Sigma Aldrich) digestion was performed in 50 mM (NH_4_)_2_CO_3_ overnight at 37 °C. The solution after proteolysis were spotted in quadruplets onto the 4-chloro-α-cyanocinnamic acid matrix (Sigma Aldrich; 3 mg mL^−1^ in TFA-acidified 50% acetonitrile), and the *m*/*z* ratios were analysed on a Thermo Fisher MALDI-LTQ Orbitrap XL spectrometer.

## Supplementary Materials

Supplementary materials can be found at https://www.mdpi.com/1422-0067/21/21/7953/s1. Table S1: Data collection and refinement statistics of reported crystal structures; Table S2: Compounds with highest measured ΔTm values in the thermal stability based assay against ERK3; Table S3: Top hits of cellular NanoBRET screening; Figure S1. Dimeric ERK3 in the crystal structure; Figure S2: Raw data of compound characterization by ITC and NanoBRET; Figure S3:. Intact mass spectra to test for full covalent adduct formation; Figure S4.: Structural superimposition between the CAF052- and GW807982X-CLK1 complexes.

## Abbreviations

ERK3	Extracellular signal-regulated kinase 3
MAPK6	Mitogen-activated protein kinase 6
ERK2	Extracellular signal-regulated kinase 2
TxY	Threonine–x–tyrosine
SEG	Serine–glutamate–glycine
SRP	Serine–arginine–proline
APE	Alanine–proline–glutamate
MAP2K	Mitogen-activated protein kinase kinase
