# Supplementary material for: Crystal Structure and Inhibitor Identifications Reveal Targeting Opportunity for the Atypical MAPK Kinase ERK3
Source: Int J Mol Sci. 2020 Oct 26;21(21):7953. doi: 10.3390/ijms21217953 (PMC7663056; doi:10.3390/ijms21217953)
Supplement: Supplementary file 1 [file ijms-21-07953-s001.pdf]

Supplementary Materials

Table S1: Data collection and refinement statistics of reported crystal structures.

**Complex**	**ERK3**	**CLK1-CAF052**
PDB Accession Code	7aqb	7ak3
Beamline	SLS X10SA	Diamond I04
	Data Collection	
Resolution[a] (Å)	50.00-2.25 (2.32-2.25)	47.96-2.50 (2.60-2.50)
Spacegroup	$P4_32_12$	$C2$
Cell dimensions	a = 83.1, b = 83.1, c = 181.2 Å	a = 91.0, b = 64.3, c = 78.5 Å
	$\alpha = \beta = \gamma = 90.0^\circ$	$\alpha = \gamma = 90.0^\circ$; $\beta = 119.3^\circ$
No. unique reflections [a]	31,101 (2,799)	13,502 (1,527)
Completeness[a] (%)	100.0 (100.0)	98.0 (98.1)
I/σI [a]	10.1 (2.1)	7.5 (1.8)
R_{merge} [a]	0.156 (2.484)	0.061 (0.468)
CC1/2 [a]	0.857 (0.562)	0.995 (0.880)
Redundancy[a]	10.7 (10.2)	2.3 (2.3)
	Refinement	
No. atoms in refinement (P/L/O) [b]	4,137/-/86	2,688/30/19
B factor (P/L/O) [b] ($Å^2$)	57/-/51	81/55/69
R_{fact} (%)	22.2	21.08
R_{free} (%)	25.5	25.99
rms deviation bond[c] (Å)	0.008	0.004
rms deviation angle[c] (°)	1.5	1.3
	Molprobity Ramachandran	
Favour (%)	97.83	94.33
Outlier (%)	0	0.30

[a] values in brackets are for high resolution shell. [b] P/L/O indicates protein/ligand in the binding pocket/others (solvent and water molecules). [c] rms indicates root-mean-square.

Table S2. Compounds with highest measured ΔTm values in the thermal stability based assay against ERK3. The ΔTm values and standard deviation for follow up are the average of two repeats.

Compound Name	ΔTm (°C) Initial Screen	ΔTm (°C) Follow Up	SMILES
CAF078	3.83	3.3 ± 0.2	CN1CCN(c2ccc(Nc3nccc(-c4cnn5ncccc45)n3)cc2C#N)CC1
AT9283	3.08	3.1 ± 0.1	C1CC1NC(Nc1c[nH]nc1c1nc2cc(CN3CCOCC3)ccc2[nH]1)=O
GW779439X	2.84	2.3 ± 0.3	CN1CCN(CC1)c1ccc(cc1C(F)(F)F)Nc1nccc(c2cnn3c2cccn3)n1
CAF052	2.81	2.5 ± 0.3	CN1CCN(c2ccc(Nc3nccc(-c4cnn5ncccc45)n3)cc2F)CC1
GW814408X	2.55	2.5 ± 0.2	COc1cccc(c1)-c1c[nH]c2c(N\N=C\c3ccncc3)ncnc12
Milcilib	2.11	2.5 ± 0.3	CNC(=O)c1nn(C)c2c1C(C)(C)Cc1cnc(Nc3ccc(N4CCN(C)CC4)cc3)nc1-2
Canertinib	2.05	3.1 ± 0.3	C=CC(=O)Nc1cc2c(Nc3ccc(F)c(Cl)c3)ncnc2cc1OCCCN1CCOCC1
XL147	1.96	1.2 ± 0.6	CC(C)(C(=O)NC1=CC(=CC=C1)S(=O)(=O)NC2=NC3=CC=CC=C3N=C2NC4=C(C=CC(=C4)OC)Cl)N
CD532	1.7	1.0 ± 0.1	C(=O)(NC1=CC=C(C=C1)NC2=NC=CC(=N2)NC3=NNC(=C3)C4CCCC4)NC5=CC=CC(=C5)C(F)(F)F
IKK-16	1.67	1.7 ± 0.4	O=C(C1=CC=C(NC2=NC=CC(C3=CC4=CC=CC=C4S3)=N2)C=C1)N5CCC(N6CCCC6)CC5
CAF045	1.61	1.4 ± 0.5	CN1CCN(c2ccc(Nc3nccc(-c4cnn5ncccc45)n3)cc2)CC1
GSK1070916	1.45	0.1 ± 0.3	CCn1cc(c2ccnc3c2cc(c2cccc(CN(C)C)c2)[nH]3)c(c2ccc(cc2)NC(N(C)C)=O)n1
GW5074	1.36	n/a	C(=C1C(Nc2ccc(cc12)I)=O)c1cc(c(c(c1)[Br])O)[Br]
GW743024X	1.32	1.2 ± 0.3	CC(=CC=C(NC(=O)C(C=CO1)=C1)C1)C=1C(C=CC1C(=O)NCC(C2)C2)=CC=1
GW784307A	1.3	n/a	[Cl].COC(C=CC=C1N(N=C2)C3=C2C(NN=CC(=CC=C2C(=O)NCCN(C)C)C=C2)=NC=N3)=C1
IMD0354	1.21	n/a	C1=CC(=C(C=C1Cl)C(=O)NC2=CC(=CC(=C2)C(F)(F)F)C(F)(F)F)O
Sorafenib	1.2	0 ± 0.5	CNC(=O)c1cc(Oc2ccc(NC(=O)Nc3ccc(Cl)c(C(F)(F)F)c3)cc2)ccn1
CGK733	1.17	n/a	O=C(NC(NC(=S)Nc1ccc(F)c([N+](=O)[O-])c1)C(Cl)(Cl)Cl)C(c1ccccc1)c1ccccc1
Butein	1.15	n/a	C1=CC(=C(C=C1C=CC(=O)C2=C(C=C(C=C2)O)O)O)O
Quizartinib	1.12	n/a	O=C(NC1=NOC(C(C)(C)C)=C1)NC2=CC=C(C=C2)C3=CN4C(SC5=CC(OCCN6CCOCC6)=CC=C45)=N3
GSK1326255A	1.12	−0.3 ± 0.3	CCCN1CCC(CC1)c1ccc(Nc2nc(Nc3cc(F)ccc3C(N)=O)c3cc[nH]c3n2)c(OC)c1
SB-250715	1.1	n/a	FC(=CC=C1C(N=C2)=C(N2C(CCNC2)C2)C(C=CN=C2OC(C=CC(OCO3)=C34)=C4)=N2)C=C1
Biofocus 093_0089_0063	1.07	0.2 ± 0.2	CN(C)c1ccc(Nc2cc(-c3ccccc3Oc3ccccc3)nc3ccnn23)cc1
GW305178X	1.04	1.1 ± 0.2	NS(=O)(=O)C(C=CC(NN=C(C(=O)N1)C(=C1C=C1)C2C1=NC=CC=2)=C1)=C1
GW580496A	1.04	0.8 ± 0.4	[Cl].CS(=O)(=O)CCNCC(=CC=C1C(C=CC(N=CN2)=C3C=2NC(C=CC(OCC(C=CC=C2)=C2)=C2[Br])=C2)=C3)O1
ALW-II-49-5	1.04	0 ± 0.2	Cc1ccc(C(=O)Nc2cccc(C(F)(F)F)c2)cc1Nc1ccc2occc(=O)c2c1
CNX-774	1.04	n/a	CNC(=O)C1=NC=CC(=C1)OC2=CC=C(C=C2)NC3=NC=C(C(=N3)NC4=CC(=CC=C4)NC(=O)C=C)F
ChemDiv Y200-4004	1.03	−0.3 ± 0.3	Cc1ccc(C(=O)Nc2cccc(C(F)(F)F)c2)cc1Nc1ccc2occc(=O)c2c1
Biofocus 040_0252_0062	1.02	−0.3 ± 0.3	Cc1ccc(C(=O)Nc2cccc(C(F)(F)F)c2)cc1Nc1ccc2occc(=O)c2c1

Biofocus 032_0099_0280	1.01	0.6 ± 0.4	Cc1ccc(C(=O)Nc2cccc(C(F)(F)F)c2)cc1Nc1ccc2occc(=O)c2c1

Abbreviations: ΔTm: melting temperature shift; n/a: not available.

Table S3. Top hits of cellular NanoBRET screening. Displayed are the average and standard deviation of a single dose measurement of both 10 and 5 μM. For comparison the ΔTm values measured in the DSF assay of each inhibitor are shown.

Name of Compound	Norm.BRET 10 μM (%)	Norm.BRET 5 μM (%)	ΔTm ERK3 (°C)	SMILES
AT9283	10.6 ± 1.5	16.1 ± 3.9	3.1 ± 0.1	N(C(=O)NC1C(=NNC=1)C2NC3C(N=2)=CC(=CC=3)CN4CCOCC4)C5CC5
JNJ-7706621	17 ± 0.1	23.1 ± 0.1	0.2 ± 0.1	C1(=CC=C(C=C1)NC2N=C(N(N=2)C(C3=C(C=CC=C3F)F)=O)N)S(N)(=O)=O
IKK-16 (IKK Inhibitor VII)	19.6 ± 5.7	28.8 ± 2.4	1.7 ± 0.4	C1(C=CC(=CC=1)NC2N=C(C=CN=2)C3=CC4C(S3)=CC=CC=4)C(=O)N5CCC(CC5)N6CCCC6
Milciclib (PHA-848125)	26.8 ± 0.5	40.9 ± 0	2.5 ± 0.3	C12CC(C3=C(C(=NC(=NC=1)NC4=CC=C(C=C4)N5CCN(CC5)C)2)N(N=C3C(NC)=O)C)(C)C
TWS119	28.1 ± 5.5	54.2 ± 6.4	0.6 ± 0.2	C1=CC=C(C=C1O)OC2=NC=NC3=C2C=C(N3)C4=CC=CC(=C4)N
GSK1070916	31.3 ± 0.1	56.8 ± 2.8	0.1 ± 0.3	C1=CN=C2C(=C1C3=CN(N=C3C4=CC=C(C=C4)NC(N(C)C)=O)CC)C=C(N2)C5=CC=CC(=C5)CN(C)C
WHI-P154	36.2 ± 6.3	50.8 ± 4.5	−0.1 ± 0.2	N1C=NC(=C2C=C(C(=CC=12)OC)OC)NC3C=C(C(=CC=3)O)Br
TG101209	36.9 ± 0.7	46.6 ± 2.2	0.3 ± 0.2	C1=C(C=C(C=C1)NC2=NC(=NC=C2C)NC3=CC=C(C=C3)N4CCN(CC4)C)S(NC(C)(C)C)(=O)=O
AEE788 (NVP-AEE788)	38.8 ± 3.1	40 ± 1.6	0.4 ± 0.3	C1=NC(=C2C(=N1)NC(=C2)C3=CC=C(C=C3)CN4CCN(CC4)CC)N[C@@H](C5=CC=CC=C5)C
AG-1478 (Tyrphostin AG-1478)	41.7	73.5 ± 1.1	0.06	C1=C2C(=CC(=C1OC)OC)C(=NC=N2)NC3=CC(=CC=C3)Cl
IPA-3	45.5 ± 4.2	67.8 ± 10.6	−3.92 ± 0.3	S(SC1C(=CC=C2C=CC=CC=12)O)C3C(=CC=C4C=CC=CC=34)O
BI-D1870	49.4 ± 1.1	80.6 ± 6.7	0.02	C1(=C(C=C(C=C1F)NC2=NC3=C(C=N2)N(C(C(N3CCC(C)C)C)=O)C)F)O
PF-00562271	49.5 ± 4.6	76.8 ± 9.4	−0.09	C1(=NC(=C(C=C1)CNC2=C(C=NC(=N2)NC3=CC=C4C(=C3)CC(N4)=O)C(F)(F)F)N(C)S(=O)(C)=O).C5=CC=CC(=C5)S(O)(=O)=O
SNS-032 (BMS-387032)	49.8 ± 4.1	71.7 ± 7.8	0.15	C1(=CN=C(O1)CSC2=CN=C(S2)NC(=O)C3CCNCC3)C(C)(C)C
CYC116	52.9 ± 2.8	64.9 ± 3.8	0.26	C1(=NC=CC(=N1)C2SC(=NC=2C)N)NC3=CC=C(C=C3)N4CCOCC4
BX-912	53.2 ± 1.7	74.1 ± 1.5	-0.12	N1(CCCC1)C(=O)NC2C=C(C=CC=2)NC3N=C(C(=CN=3)Br)NCCC4=CN=CN4
Vandetanib (ZD6474)	56.9 ± 4.1	69 ± 0.5	0.2	C12=C(C(=NC=N1)NC3=C(C=C(C=C3)Br)F)C=C(C(=C2)OCC4CCN(CC4)C)OC
ENMD-2076	58.5 ± 0.4	90.8 ± 3	−0.15	C1(N=C(C=C(N=1)N2CCN(CC2)C)NC3=NNC(=C3)C)/C=C/C4C=CC=CC=4
PRT062607 (P505-15, BIIB057) HCl	60.1 ± 16.9	88.2 ± 10.3	0.51	N1(=C(N=C(C(=C1)C(N)=O)NC2=CC(=CC=C2)N3N=CC=N3)N[C@@H]4CCCC[C@H]4N).Cl
AZD5438	61.7 ± 2.2	93.3 ± 4	−0.17	C1=C(C=CC(=C1)NC2=NC=CC(=N2)C3=CN=C(N3C(C)C)C)S(=O)(C)=O

Abbreviations: Norm.BRET: normalised BRET; ΔTm: melting temperature shift; n/a: not available.

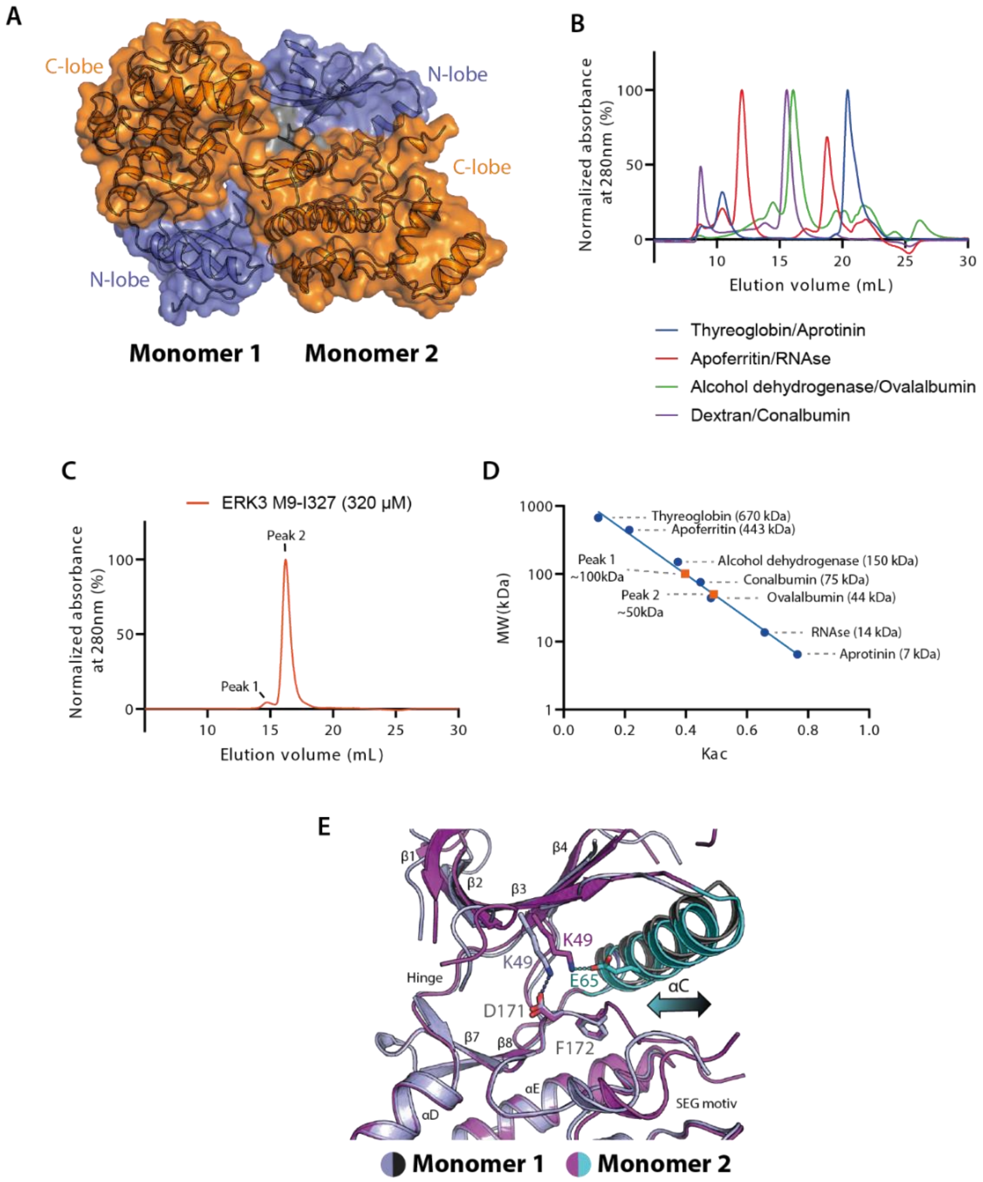

Figure S1. Dimeric ERK3 in the crystal structure. (**A**) Face-to-face, head-to-tail dimer of ERK3 in the asymmetric unit. The kinase *N*-terminal and *C*-terminal lobes are in blue and orange, respectively. (**B**) Size exclusion chromatograms of protein markers. (**C)** Size exclusion chromatogram of ERK3 at 320 μM. (**D)** The standard curve calculated from the elution profiles of protein markers. Kav values of each marker were calculated from the equation: Kav = (Ve − Vo) / (Vt − Vo), where Ve = elution volume, Vo = void volume and Vt = column volume, and plots against protein molecular weight in kDa in logarithmic scale. Organe dots in the plots are the Kav values for ERK3 from both elution points with their estimated molecular weight calculated based on the standard curve. (**E**) Superimposition reveals highly conserved structure of both ERK3 monomers present in the asymmetric unit.

A

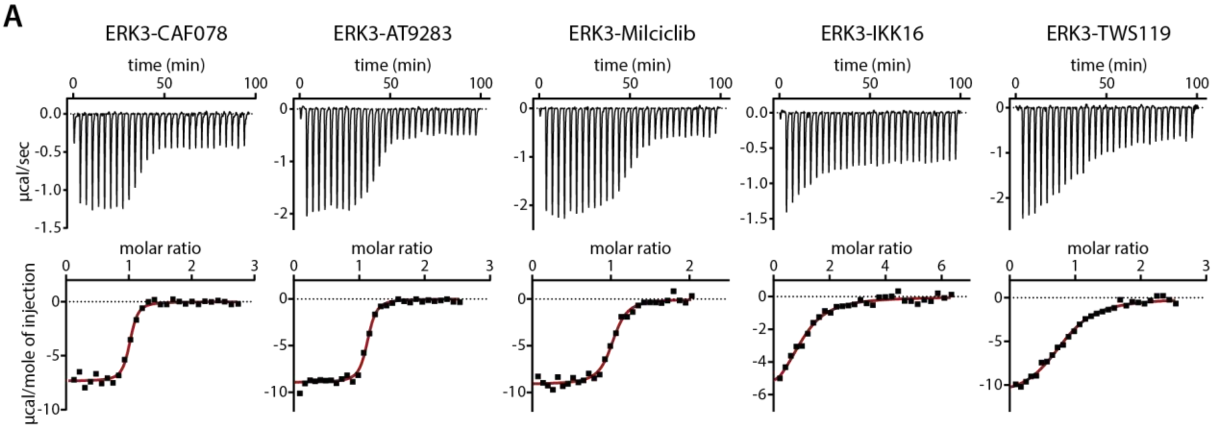

B

	CAF078	AT9283	GW779439X	Milciclib	IKK-16	TWS119
Kd (µM)	0.044 ± 0.026	0.080 ± 0.005	0.115 ± 0.038	0.245 ± 0.060	1.848 ± 0.915	1.927 ± 0.182
dH (kcal/mol)	-7.622 ± 0.379	-8.905 ± 0.054	-9.049 ± 0.540	-9.386 ± 0.355	-6.196 ± 0.760	-11.460 ± 0.085
n	0.939 ± 0.054	1.040 ± 0.072	0.968 ± 0.013	0.990 ± 0.014	0.967 ± 0.061	0.852 ± 0.035
-TdS (kcal/mol)	-2.300	-0.459	-0.121	0.622	-1.40	3.924
dG (kcal/mol)	-9.922 ± 0.188	-9.364 ± 0.034	-9.170 ± 0.176	-8.724 ± 0.140	-7.567 ± 0.296	-7.537 ± 0.055

C

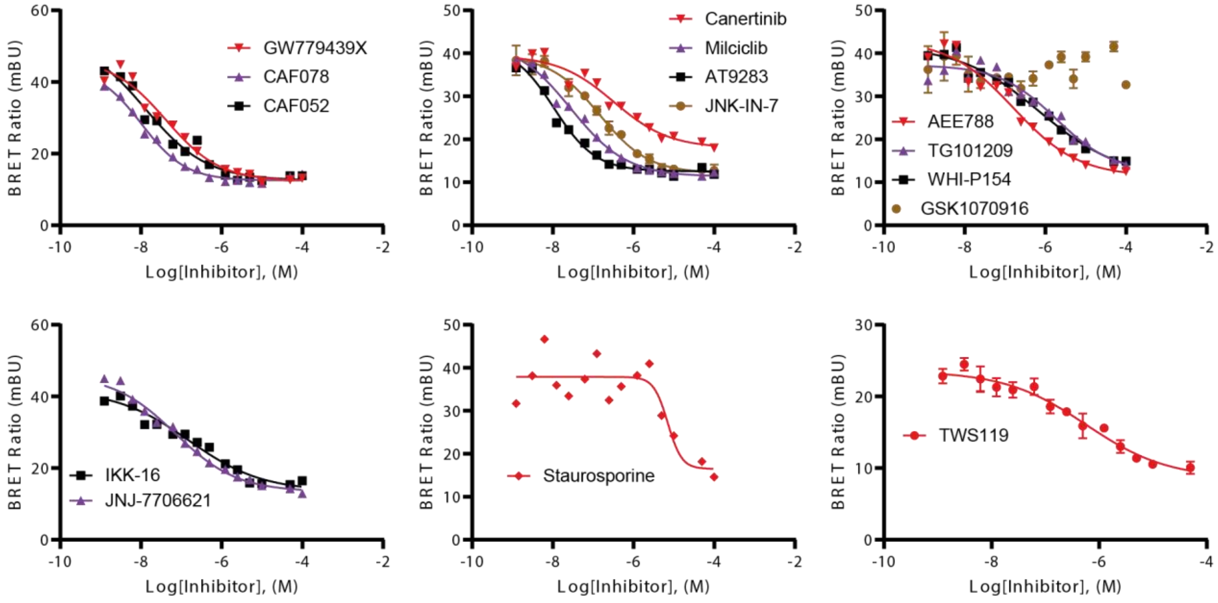

Figure S2. (**A**) ITC results of ERK3 titrated with various compounds. Shown are the raw heat rate measured over time and the integrated heat used for the binding model fit of one representative titration. (**B**) Binding affinity and thermodynamics of ERK3-inhibitors complexes. Values displayed are the average and standard deviation of at least two independent experiments (**C**) NanoBRET dose response curves of inhibitors measured in HEK293T cells. Shown is one representative titration for all analysed inhibitors.

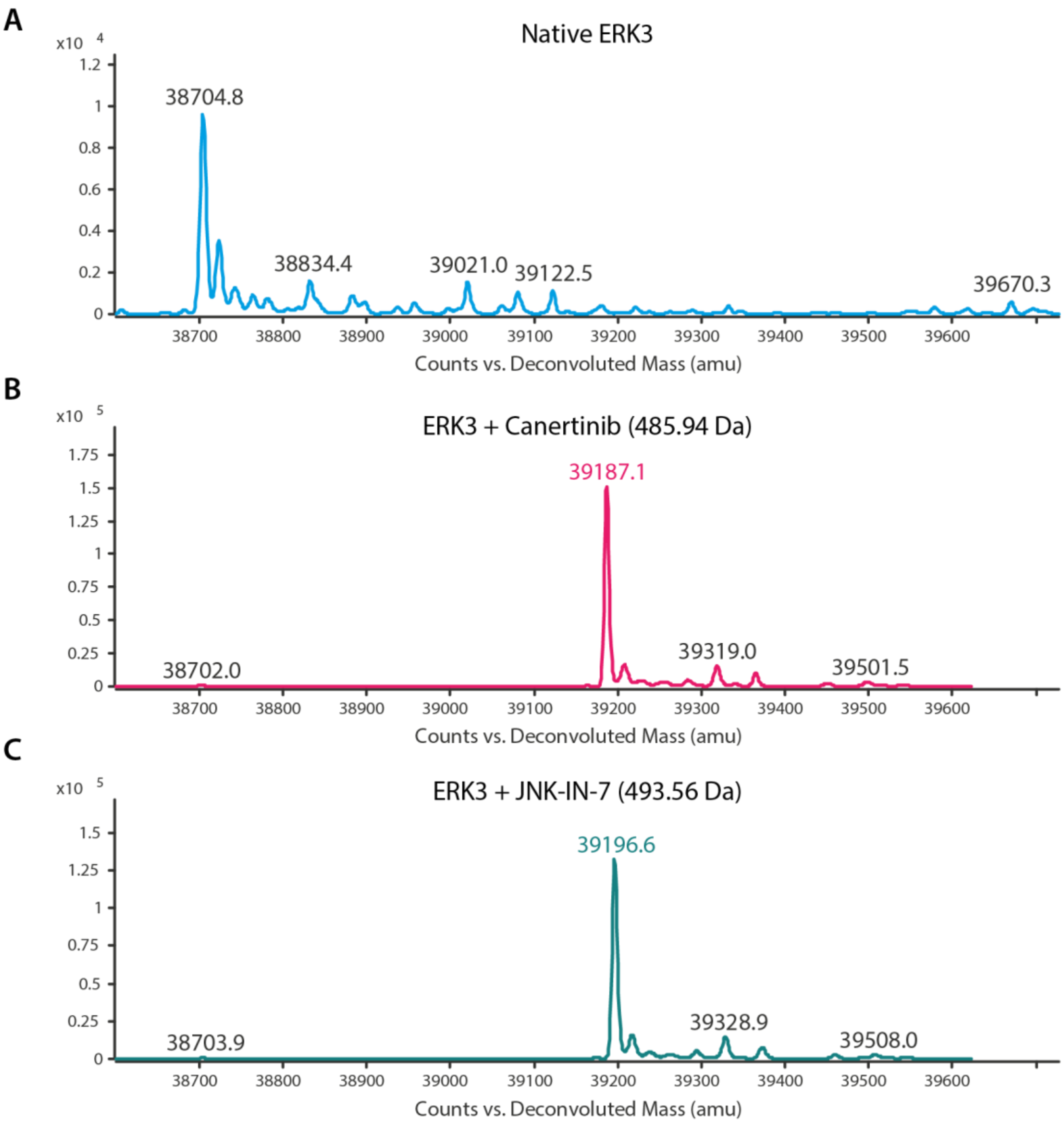

Figure S3. (**A**–**C**) Intact mass spectra to test for full covalent adduct formation. ERK3 was either incubated with DMSO (**A**), two-fold excess of canertinib (**B**) or JNK-IN-7 (**C**) for 4 h at room temperature.

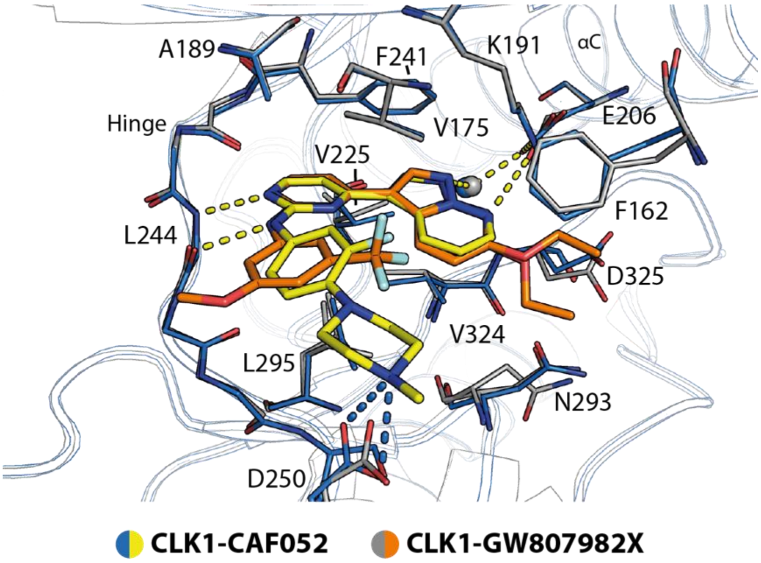

Figure S4. Structural superimposition between the CAF052- (pdb id 7ak3) and GW807982X-CLK1 complexes (pdb id 6zln) revealed highly similar binding modes of both chemically-related inhibitors in the kinase.

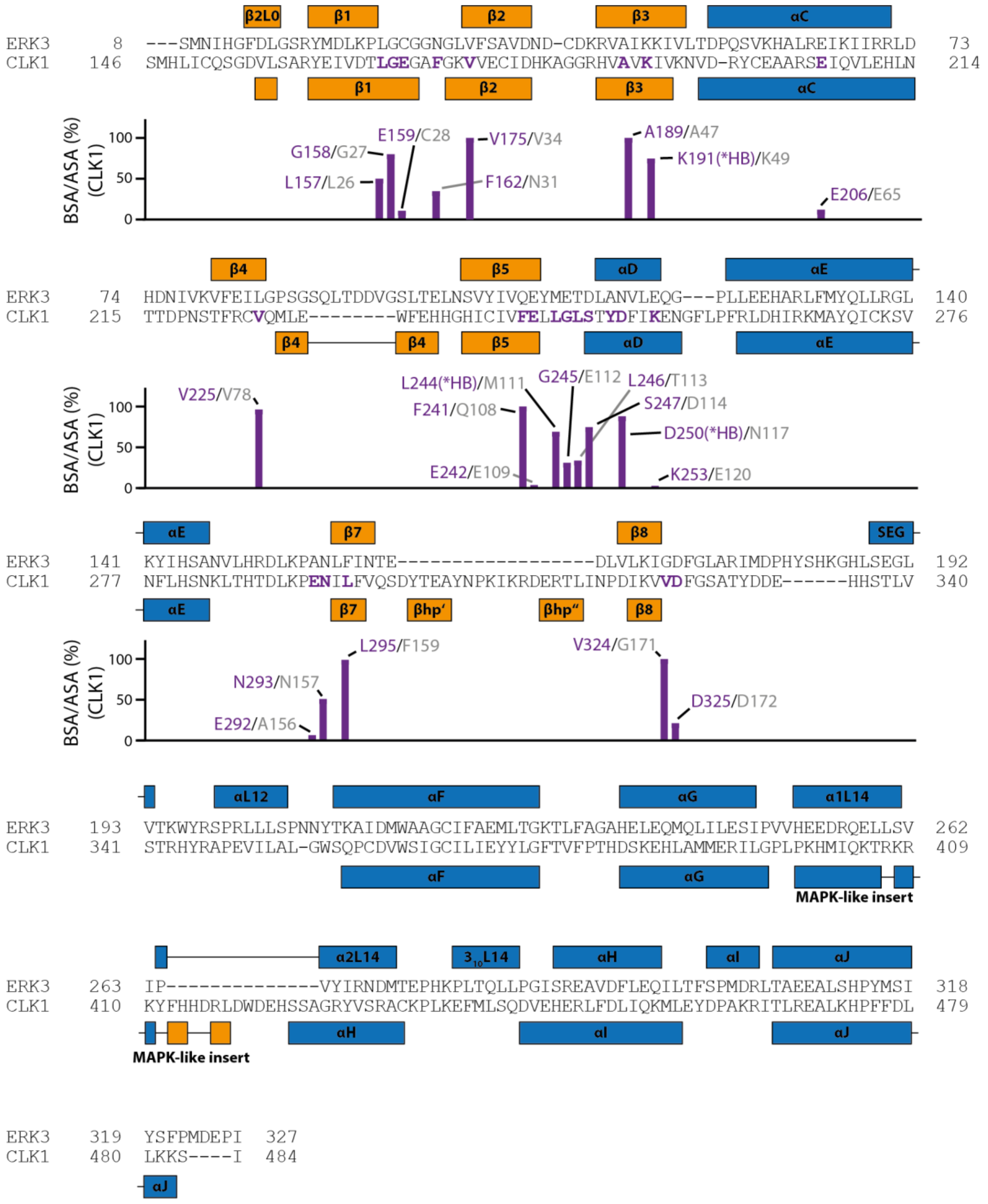

Figure S5: Structure-based sequence alignment of kinase domains of CLK1 and ERK3. Secondary structural elements (α-helices in blue and β-strands in orange) are shown for ERK3 (above) and for CLK1 (below). CLK1 residues interacting with CAF052 are highlighted in magenta. The individual contribution of each residue of CLK1 towards the complex formation with CAF052 expressed by the ratio of buried surface area (BSA)/available surface area (ASA) is shown beneath the alignment (magenta). CLK1 residues forming hydrogen-bonds with CAF052 are highlighted and denoted with "*HB". The equivalent residues in ERK3 are displayed in grey.
